# Detection of SARS‐CoV‐2 in Semen Months After COVID‐19: Potential Evidence of Low‐Level Replicative Persistence

**DOI:** 10.1111/andr.70431

**Published:** 2026-11-09

**Authors:** Marcelo H. Furtado, Fernanda R. Souza, Lidia M. Andrade, Bárbara P. Mendes, Juan C. González, Thiago P. Furtado, Anisleidy Pérez Castillo, Samyra M. S. N. Lacerda, Renato S. Aguiar, Guilherme M. J. Costa

**Affiliations:** ^1^ Cell Biology Lab Department of Morphology Institute of Biological Sciences Federal University of Minas Gerais Belo Horizonte Brazil; ^2^ Clínica MF, Belo Horizonte Belo Horizonte Brazil; ^3^ Department of Urology and Human Reproduction Mater Dei Hospital Belo Horizonte Brazil; ^4^ Department of Genetics Ecology and Evolution Institute of Biological Sciences Federal University of Minas Gerais Belo Horizonte Brazil; ^5^ Department of Physics Institute of Exact Sciences Federal University of Minas Gerais Belo Horizonte Brazil; ^6^ Department of Urology David Geffen School of Medicine UCLA Los Angeles California USA; ^7^ D'Or Institute for Research and Education (IDOR) Rio de Janeiro Brazil

**Keywords:** coronavirus infection, male genital tract, viral persistence, viral reservoirs, virus detection

## Abstract

**Background:**

Viral persistence is considered one of the key mechanisms driving long COVID symptoms. The male genital tract (MGT), particularly the testis, is an immune‐privileged site that may serve as a viral reservoir for SARS‐CoV‐2. However, the presence of SARS‐CoV‐2 in semen and its long‐term infectiousness remains controversial.

**Objectives:**

We aimed to investigate the long‐term presence and potential replicative activity of SARS‐CoV‐2 in semen, evaluating whether the virus can persist and maintain low‐level culture signals in the MGT well beyond the acute phase of infection.

**Material and Methods:**

This observational, prospective, cross‐sectional study included 38 men treated at Mater Dei Hospital, Brazil, between October 2020 and October 2021, who had recovered from mild to moderate COVID‐19 and consented to participate. Semen samples were collected from mildly symptomatic participants at approximately 1 month (15– 45 days), 3 months, and 6 months after symptom onset. The moderately symptomatic group provided a single sample approximately 6 months post‐infection. Semen analysis and SARS‐CoV‐2 detection were performed using RT‐qPCR, followed by viral isolation in Vero CCL‐81 cells. Potential viral persistence and replication signals were evaluated through immunofluorescence and transmission electron microscopy (TEM).

**Results:**

SARS‐CoV‐2 was detected by RT‐qPCR in 21 of 38 patients (55%), with high cycle threshold (Ct 38–39) values observed for more than 6 months post‐infection. Following inoculation into Vero cell cultures, a low‐level in vitro expansion of viral copies was identified in a subset of samples from mild and moderate patients. The potential presence of the virus was further supported by the presence of viral proteins via immunofluorescence and the identification of structures morphologically compatible with coronavirus‐like particles by TEM.

**Conclusion:**

This study provides evidence of the long‐term persistence of viral RNA and culture‐derived signals suggestive of restricted or low‐level replicative activity in semen. These findings suggest that the MGT may serve as a viral reservoir, highlighting the importance of longitudinal semen analysis for monitoring viral persistence and reservoir dynamics after clinical recovery.

## 1 Introduction

Long COVID is a chronic, multisystem condition that persists for at least 3 months after acute SARS‐CoV‐2 infection. It may present as a continuous, relapsing, remitting, or progressive disease and affects millions worldwide despite advances in vaccination and changes in viral virulence. The enduring impact of long COVID continues to pose a substantial burden on healthcare systems and economies globally [1].

Although the underlying mechanisms of long COVID remain incompletely understood, several pathways have been implicated, including viral persistence, immune dysregulation, mitochondrial and complement system dysfunction, prothrombotic inflammation, and alterations in the microbiome [1]. Among these, viral persistence has emerged as a particularly compelling factor. This phenomenon, documented across a range of viral infections, refers to the sustained presence of viral genetic material and/or proteins in host tissues beyond the acute phase. Mounting evidence supports an association between viral persistence and post‐acute sequelae of SARS‐CoV‐2 (PASC), suggesting it may contribute directly to the chronic symptoms’ characteristic of long COVID [1].

The testis is among several organs considered immune‐privileged sites, along with the brain, the eye, and the pregnant uterus [2]. While this status preserves the testicular function by limiting immune responses, it also creates a potential sanctuary for viral pathogens [2]. SARS‐CoV‐2 has demonstrated tropism for testicular tissue, with viral RNA and particles identified in autopsy specimens from men who died with COVID‐19 [3]. The virus has been detected in multiple testicular cell types—including Sertoli and Leydig cells, spermatogonia, and macrophages—and even within the seminiferous tubules lumen [3]. Furthermore, the blood–testicular barrier may not constitute a perfect barrier to viruses, especially in the presence of systemic or local inflammation. Notably, viral particles have been detected within the seminiferous tubules, raising the possibility of SARS‐CoV‐2 shedding in the semen [3].

Given that at least 27 different viruses have been identified in human semen [4], the possibility of SARS‐CoV‐2 shedding in semen has generated significant interest. While SARS‐CoV‐2 has been detected in testicular tissue, its presence in semen remains controversial. Most studies, with few exceptions [5, 6, 7, 8, 9, 10], have not detected SARS‐CoV‐2 in semen. However, these studies often involved limited follow‐up and variable timing of sample collection, typically focusing on the acute or early recovery phase. Indeed, most of these studies reported the persistence of SARS‐CoV‐2 based on the detection of viral genomic RNA, without investigating viral cultivability or potential replicative activity in semen.

This study aimed to fill a critical knowledge gap by investigating the long‐term presence and potential replicative activity of SARS‐CoV‐2 in semen, evaluating whether the virus can persist and maintain culture‐derived replication signals in the male genital tract (MGT) well beyond the acute phase of infection. We hypothesized that replication‐competent SARS‐CoV‐2 particles may persist in semen for extended periods following clinical recovery.

## 2 Methodology

### 2.1 COVID‑19 Patients

This observational and prospective study included adult men (18–50 years) diagnosed with COVID‐19 by RT‐PCR from oropharyngeal swabs, who attended Hospital Mater Dei between October 2020 and October 2021, and consented to participate. The only exclusion criterion was prior vasectomy. Participants were divided into two groups based on symptom severity: mildly symptomatic (not hospitalized) and moderately symptomatic (hospitalized). To evaluate both long‐term recovery and late‐stage outcomes, we employed a mixed study design: the mildly symptomatic group was followed prospectively as a longitudinal cohort (with multiple collections), whereas the moderately symptomatic group was assessed as a cross‐sectional cohort (providing a single sample at 6 months).

All participants had clinically recovered from COVID‐19. Clinical data were collected through a questionnaire, and biological samples were obtained after obtaining informed consent. The study was approved by the National Research Ethics Committee (CONEP) under CAAE: 36122420.5.0000.5128 and conducted in accordance with the Declaration of Helsinki. Access to Brazilian genetic heritage was registered in the National System for the Management of Genetic Heritage and Associated Traditional Knowledge (SISGEN), under registration number AC1D978.

### 2.2 Semen Specimens

After completing a 15‐day quarantine, mildly symptomatic patients provided semen samples at approximately 1 month (median 27 days; range 15–45 days), 3 months, and 6 months after symptom onset. Moderately symptomatic patients provided a single sample 6 months after diagnosis. Samples were collected by masturbation, after 2–3 days of abstinence, in a clinic laboratory room following strict hygiene protocols. Remaining raw semen from both groups was frozen in liquid nitrogen (−196°C) and stored in a dedicated biorepository at the MF Male Fertility Clinic under the supervision of one of the authors (MHF) until analysis.

### 2.3 Semen Analysis

Semen samples were analyzed following WHO guidelines (sixth edition, 2021) for assessing semen volume, pH, viscosity, concentration, motility, morphology, vitality, and leukocyte count. Volume was determined by weighing the collection container. Semen pH was measured using pH indicator paper (range 6.0–10.0), and viscosity was estimated by observing the length of the thread formed upon aspirating the sample into a disposable pipette. After liquefaction, concentration and motility were assessed under a phase‐contrast microscope (×200–400, OLYMPUS BX43) with standard dilutions applied when necessary. Sperm motility was evaluated using a Makler chamber with a 10 µL aliquot, analyzing at least 200 spermatozoa. Sperm vitality was evaluated using eosin–nigrosin staining to determine the proportion of live spermatozoa. Morphology was assessed using Tygerberg's strict criteria after Papanicolaou staining, under oil immersion (×100). A single experienced technician performed all analyses.

### 2.4 Lymphocytic Panel

We used flow cytometry (FACSCalibur, BD Biosciences, USA) to detect the presence of CD3, CD4, CD8, and CD45 cells in semen. In brief, round cells were obtained by centrifuging the semen sample in a 40% density gradient at 1200 RPM for 15 min. After centrifugation, the formed central halo was separated from the gradient using a Pasteur pipette, washed twice in PBS 1×, and centrifuged at 1200 RPM for 7 min. Subsequently, the cells were quantified in a Neubauer chamber.

Cells were stained with anti‐CD45, CD3, CD4, and CD8 monoclonal antibodies using the BD Multitest CD3/CD8/CD45/CD4 kit. One million cells were incubated in the dark for 15 min at room temperature, washed, and then resuspended in 500 µL of 1× PBS. Data acquisition (20,000 events) was performed on a BD FACSCalibur and analyzed using BD CellQuest software. Based on FSC versus SSC profiles, T lymphocytes (CD3+) were identified within the CD45+ gate and characterized as CD4+ or CD8+ (Figure S1).

### 2.5 Detection of SARS‑CoV‑2 Genetic Material in Semen

#### 2.5.1 SARS‑CoV‑2 Detection Using RT‑qPCR

Semen samples were analyzed in their raw, unprocessed form and were stored in an ultra‐freezer at −80°C until RT‐qPCR analysis. No density gradient separation or centrifugation was performed prior to RNA extraction, in order to preserve the native composition of the ejaculate and avoid potential loss of viral material during processing steps.

We used two protocols for RT‐qPCR. For the first cohort of 15 patients (all mildly symptomatic), total RNA was extracted using the QIAamp Viral RNA Mini Kit (Qiagen), following the manufacturer's instructions. The presence of SARS‐CoV‐2 viral RNA was detected by RT‐qPCR using primers to amplify the envelope (E) gene and the human transcript of the RNAse P (RNP) gene as the endogenous control.

To enhance analytical sensitivity for the detection of low viral loads, the protocol was subsequently optimized for the remaining cohort of 23 patients (comprising 12 mild and 11 moderate cases). In this context, we extracted the total RNA following the protocol outlined in the PureLink RNA Mini Kit (Invitrogen, USA). A water‐only control (RNA Blank) was included with each round of extraction to check for RNA contamination. The RT‐qPCR reaction was performed using iTaq Universal Probes One‐Step Kit (Bio‐Rad). The nucleocapsid gene's N1 locus of the SARS‐CoV‐2 RNA was measured with a high‐precision primer‐probe set (IDT, Coralville, USA), selected for its superior performance in detecting viral copies near the assay's limit of detection (LOD). The human transcript of the 18S ribosomal RNA (18S rRNA) was used as the endogenous control, ensuring the reliability of the results.

Reactions were run on the CFX96 Touch Real‐Time PCR Detection System (Bio‐Rad Laboratories, Hercules, CA) under the following conditions: reverse transcription at 50°C for 10 min followed by a single cycle of 95°C for 3 min, then 45 cycles of PCR with denaturation at 95°C for 15 s, annealing at 55°C for 30 s and extension at 60°C for 30 s. Samples were considered positive with a cycle threshold (Ct) ≤ 40.

#### 2.5.2 SARS‐CoV‐2 Whole‐Genome Sequencing

Libraries were prepared using the QIAseq FX DNA Library Prep kit (QIAGEN, Hilden, NRW, Germany). Genomes were sequenced on the MiSeq platform (Illumina, San Diego, CA, USA) using v3 cartridges (600 cycles) according to the manufacturer's instructions. Three negative controls were used at all sample processing steps (cDNA synthesis, viral genome amplification, and library preparation) in each batch.

#### 2.5.3 Viral Genome Assembly and Classifications

Sequencing data were processed with a custom pipeline. First, a quality control step was performed with Trimmomatic v0.39, which removed adapter and primer sequences, short reads (<50 nucleotides), and low‐quality bases (Phred score < 30). Then, reads were mapped to the SARS‐CoV‐2 reference genome (GenBank accession: NC_045512) with Minimap2, and unmapped reads were discarded. Mapping files were then indexed and sorted with SAMtools v1.12, while the BCFtools v1.12, consensus option was used to estimate consensus genome sequences. Low‐coverage sites were masked with BEDtools v2.30.0. Depth thresholds sites with less than 10‐fold depth were masked. Consensus sequences were then classified using the Pangolin tool v.3.1.11 or the NextClade web application v.1.7.0.

### 2.6 SARS‑CoV‑2 Isolation

#### 2.6.1 Vero Cell Culture

In a BSL‐3 lab, 1×10^5^ Vero cells (ATCCCCL‐81) in 500 µL of Dulbecco’s Modified Eagle Medium (DMEM) were seeded into 24‐well plates and incubated at 37°C with 5% CO_2_ for 24 h until 90% confluence. Only samples that tested positive for SARS‐CoV‐2 were used for inoculation. Prior to inoculation, all semen samples underwent RT‐qPCR analysis. Only samples that tested positive for the SARS‐CoV‐2 N‐gene were used for inoculation. Prior to inoculation, all semen samples underwent RT‐qPCR analysis. To evaluate viral isolation and culture reproducibility, a total of 24 specimens were selected for inoculation, comprising 12 positive specimens from the mildly ill group (obtained from 10 patients, including longitudinal collections from two patients who tested positive at multiple time points) and 12 specimens from the moderately ill group. The latter consisted of 10 primary positive specimens from 10 patients, plus 2 redundant parallel specimens (representing duplicate aliquots) to assess the reproducibility of the culture system.

The samples were thawed and diluted 1:10 in DMEM. Subsequently, 100 µL of the diluted semen was added to each well and incubated for 1 h at 37°C with gentle intermittent mixing to facilitate viral adsorption. Following incubation, 1 mL of fresh DMEM was added, and the cells were maintained at 37°C and 5% CO_2_ for 72 h. Supernatants (150 µL) were collected every 24 h, mixed with lysis buffer plus beta‐mercaptoethanol, and stored at −80°C for subsequent RNA extraction and RT‐qPCR. Each sample yielded three time‐point specimens. Negative controls consisted of uninoculated Vero cells in DMEM alone, which consistently showed no cytopathic effects and no RT‐qPCR amplification throughout the 72‐h culture period. Positive controls used DMEM containing the SARS‐CoV‐2 Delta strain (MOI 0.01) and demonstrated rapid cytopathic effects accompanied by a continuous, exponential increase in viral copy numbers (decrease in Ct values) over 72 h. In patient cultures, potential viral activity in supernatants was evaluated based on the trend of RT‐qPCR Ct values over 72 h.

Viral loads in culture supernatants were quantified by converting RT‐qPCR cycle threshold (Ct) values into viral RNA copy numbers per mL. Viral counts (Log10) were automatically calculated by the CFX Manager Software (Bio‐Rad) based on a standard curve, generated through serial dilutions of a SARS‐CoV‐2 sample of the known concentration. The software converted cycle threshold (Ct) values into absolute quantities using the linear regression equation *y* = −3.625*x* + 42.625, where y represents the Ct value and *x* the Log_10_ virus count.

#### 2.6.2 Immunofluorescence in Vero Cells

Vero cell infection was confirmed via immunofluorescence using a commercial anti‐S protein antibody (Abcam, USA). Cells were seeded in 96‐well black plates with transparent bottoms and incubated with 50 µL of third‐passage supernatant for 1 h at 37°C and 5% CO_2_ with homogenization. Then, 50 µL of DMEM was added and incubated for 24 h.

Cells were fixed with 4% paraformaldehyde for 15 min, washed with 1× PBS, and permeabilized with 1% PBS‐Triton for 15 min. Blocking was performed with PBS containing 5% Donkey Serum for 1 h. Cells were then incubated with rabbit anti‐S protein antibody (1:1000) for 1 h at 37°C, followed by Alexa Fluor 488‐conjugated donkey anti‐rabbit secondary antibody (1:1000, Invitrogen) for 2 h at room temperature in the dark. Nuclei were stained with DAPI (300 nM) for 15 min.

After washing, fluorescence images were captured using the BioTek Cytation 5 system. The signal was measured at an excitation of 495 nm and an emission of 520 nm. Negative controls included samples incubated with either the primary or the secondary antibody alone.

#### 2.6.3 Transmission Electron Microscopy in Vero Cells

The transmission electron microscopy (TEM) was used to confirm SARS‐CoV‐2 infection in Vero cells. Cells were detached with trypsin, washed in 0.05 M phosphate buffer, and fixed in 4% glutaraldehyde for 4 h. After rinsing, the pellets were post‐fixed in reduced osmium (1% OsO_4_ + 1.5% potassium ferrocyanide), dehydrated in ethanol, and embedded in Araldite resin.

Ultrathin sections (60 nm) were cut with a diamond knife on a Leica EM UC6 ultramicrotome (Leica Microsystems), mounted on 200‐mesh copper grids (Ted Pella), stained with lead citrate (Merck, USA), and examined under a Tecnai G2‐12 transmission electron microscope (Thermo Fisher/FEI). Viral particles were identified by their 60–140 nm round or oval shape, membrane envelope, electron‐dense core, and localization within membrane‐bound compartments.

### 2.7 Statistical Analysis

Descriptive statistics were used: mean ± SD for normally distributed data; median and interquartile range (IQR) for non‐normally distributed data; and frequencies for categorical variables. The Wilcoxon rank‐sum test was used to compare medians between non‐normal groups, and the Friedman *Q* test was used for repeated non‐parametric measures. Post hoc comparisons employed the Conover test with Bonferroni correction. Fisher's exact test was used to assess categorical variables. A *p*‐value < 0.05 was considered significant. Analyses were performed using Stata 17 (StataCorp, College Station, TX, USA).

## 3 Results

### 3.1 Patient's Symptoms and Sample Collection

Of the 38 study participants, 27 had mild COVID‐19, and 11 had moderate disease. Only one mild‐case patient reported testicular symptoms, and four participants had infertility risk factors, including smoking, anabolic steroid use, previous varicocelectomy, and cryptorchidism (Table 1).

**TABLE 1:** Baseline characteristics and study design.

			All	Mild	Moderate
*N*			38	27	11
Age (years, mean, ±SD; range)			38.3±6.4 (28–52)	38.5±6.8 (28–52)	37.9±5.7 (31–48)
Testicular pain (*n*(%))			1/38 (2.6%)	1/27 (3.7%)	0/11 (0%)
Smoke			1/38 (2.6%)	1/27 (3.7%)	0/11 (0%)
Infertility factor			3/38 (7.9%)	2/27 (7.4%)^*^	1/11 (9.1%)^**^
Vaccination^***^			20/38 (52.6%)	11/27 (40.7%)	9/11 (81.8%)
Semen collection	1 month	*n* (%)		27/27 (100%)^$^	
		days, median (IQR)		27 (21–30)	
	3 months	*n* (%)		12/27 (44.4%)^&^	
		days, median (IQR)		129 (122–142)	
	6 months	*n* (%)		8/27 (29.6%)^@^	11/11 (100%)
		days, median (IQR)		221 (218–221)	200 (184–239)

*Notes*:

^$^ 14 only 1st collection.

^&^ 5 only 1st and 2nd collection.

^@^ 1 collected only the 1st and 3rd.

* Varicocelectomy and criptorchidism.

** Anabolic steroids use.

*** At least 1 dose of vaccine.

In the mild group, 46 semen samples were collected. The first sample (27 patients) was obtained on average 27 days post‐symptom onset. Follow‐up collections occurred in 40.7% and 29.6% of patients, at approximately 129 and 221 days, respectively. All moderate‐group patients provided one sample approximately 200 days after symptom onset (Table 1).

### 3.2 Seminal Parameters and Lymphocytic Profile

In patients with mild COVID‐19, there was a non‐significant trend toward reduced sperm count, concentration, and total motility in the second sample, followed by recovery in the third. Notably, leukocyte concentration decreased in the third sample (Table 2). Lymphocyte markers (CD3, CD4, CD8, and CD45) tended to increase in the second sample and then declined below baseline in the third (Table 3).

**TABLE 2:** Semen parameters.

	Collection (months)	All^*^	Mild	Moderate^*^
Volume (mL, median (IQR))	1	—	3.0 (2.3–4.0)	—
	3	—	3.4 (1.8–3.8)	—
	6	3.3 (2.3–4.0)	2.9 (2.3–5.1)	3.4 (2.3–4.0)
Count (millions, median (IQR))	1	—	210.6 (64.0–317.3)	—
	3	—	139.7 (89.3–220.0)	—
	6	216.9 (101.2–363.0)	185.9 (91.0–285.7)	255.6 (123.2–533.5)
Concentration (millions/mL, median (IQR))	1	—	69.3 (18.2–96.8)	—
	3	—	51.2 (38.2–57.9)	—
	6	63.9 (44.0–97.3)	58.4 (38.1–87.1)	68.8 (50.0–136.8)
Total motility (%, median (IQR))	1	—	62.0 (39.0–73.0)	—
	3	—	60.0 (53.0–79.0)	—
	6	76.5 (73.0–81.0)	74.5 (60.5–79.0)	80.0 (74.0–87.0)
Progressive motility^**^ (%, median (IQR))	1	—	51.0 (33.0‐63.0)	—
	3	—	51.5 (43.0‐70.0)	—
	6	71.0 (64.0–77.0)	68.5 (52.5–71.5)	75.5 (68.0–82.0)
Morphology (%, median (IQR))	1	—	3 (2–5)	—
	3	—	4 (4–6)	—
	6	4 (4–5)	4 (4–5)	4 (3–6)
Round Cells (millions/ml, median (IQR))	1	—	3.0 (1.9–7.6)	—
	3	—	3.0 (1.8–4.5)	—
	6	2.2 (1.2‐5.5)	1.7 (1.1–6.7)	2.6 (1.4–5.1)
Leukocytes (millions/ml, median (IQR))	1	—	0.3 (0.1–1.3)	—
	3	—	0.3 (0.04–0.8)	—
	6	0.1 (0.05–0.2)	0.15 (0.06–0.2)	0.1 (0.04–0.2)
Any seminal abnormality^***^ (n, %)	1	—	20/27 (74.1)	—
	3	—	5/12 (41.7)	—
	6	5/18 (27.8)	2/8 (25.0)	3/10 (30.0)

*Notes*: Semen vitality, pH, and viscosity remained within normal WHO reference ranges with no abnormalities or significant differences.

* Excluded one patient on anabolic steroids use.

** Sum of rapidly progressive (grade a) and slowly progressive (grade b) spermatozoa.

*** Volume, count, concentration, total motility, progressive motility, morphology, leukocytes.

**TABLE 3:** Lymphocytic profile.

	Collection (months)	All^*^	Mild	Moderate^*^
CD3 (%, median (IQR))	1	—	1.36 (0.96–3.15)	—
	3	—	2.15 (1.06–3.25)	—
	6	0.36 (0.22–0.80)	0.36 (0.23–2.97)	0.32 (0.11–0.61)
CD4 (%, median (IQR))	1	—	0.60 (0.34–1.66)	—
	3	—	1.21 (0.73–2.89)	—
	6	0.20 (0.13–0.44)	0.21 (0.13–2.08)	0.18 (0.08–0.32)
CD8 (%, median (IQR))	1	—	0.84 (0.40–2.18)	—
	3	—	1.19 (0.63–1.84)	—
	6	0.02 (0.01–0.03)	0.02 (0.01–2.15)	0.02 (0.01–0.03)
CD45 (%, median (IQR))	1	—	4.58 (2.61–8.13)	—
	3	—	5.06 (1.73–10.74)	—
	6	1.72 (1.15–5.07)	1.58 (1.15–5.07)	2.21 (1.08–6.54)

*Note*:

* Excluded one patient on anabolic steroids use.

Among the seven patients who provided all three samples (representing a longitudinal cohort of 7 completers out of 27 initially recruited), no significant changes were found, although the same trends were observed (Figure 1). Given this limited sample size and the high attrition rate, these longitudinal comparisons are exploratory and hypothesis‐generating. Patients with moderate COVID‐19 showed no abnormalities in semen parameters around 200 days post‐symptom onset (Table 2).

**FIGURE 1:**
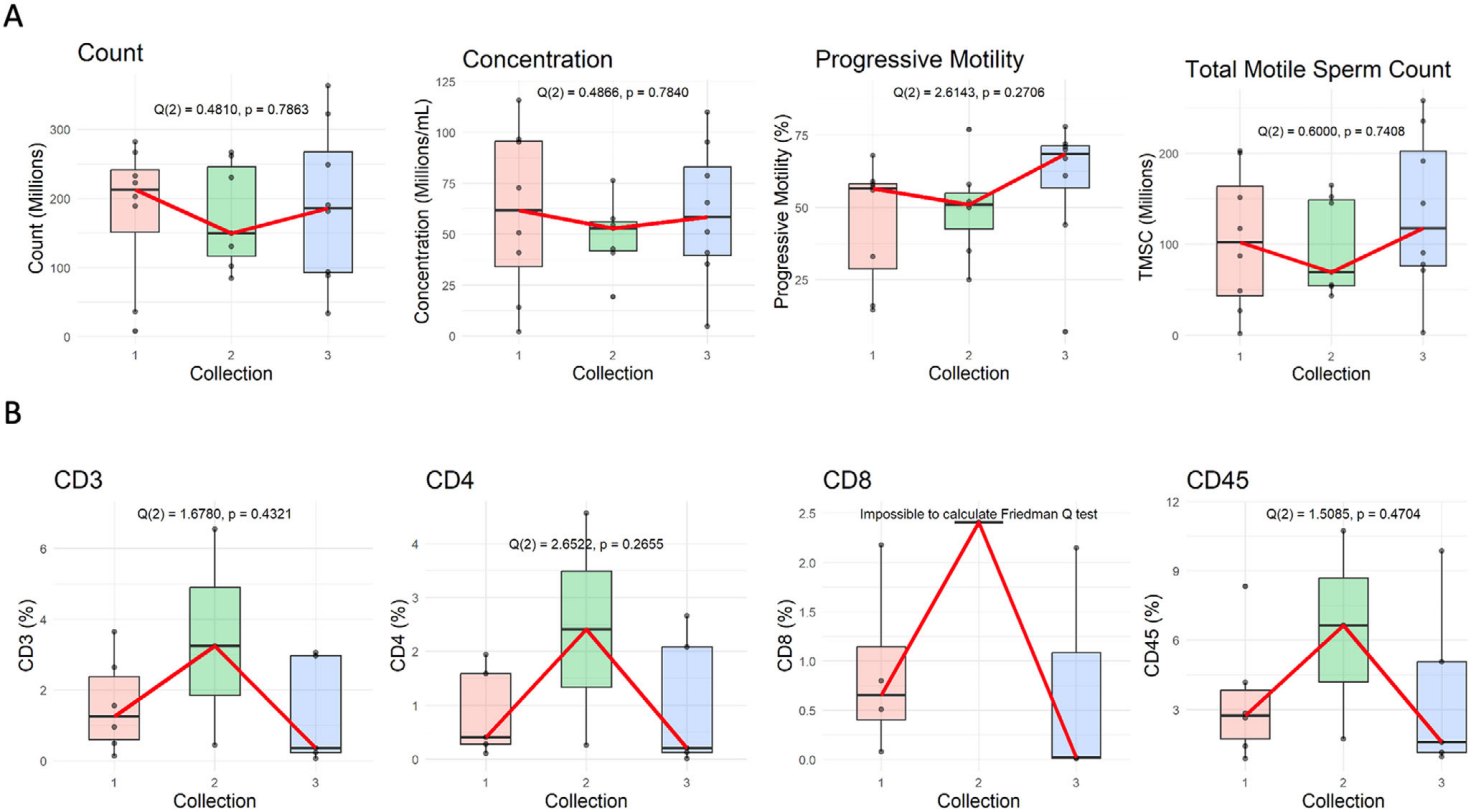
Longitudinal profiles of seminal parameters and lymphocytic subpopulations in patients recovered from mild COVID‐19. (A) Semen parameters (sperm count, concentration, progressive motility, and total motile sperm count) and (B) lymphocyte percentages (CD3+, CD4+, CD8+, and CD45+) across the three consecutive collection points (nominal 1, 3, and 6 months post‐infection). Box plots represent the median, interquartile range (IQR), and range. The red trend lines indicate the mean values. Given the small longitudinal sample size (7 completers out of 27 patients initially recruited), these results are exploratory and hypothesis‐generating. Repeated‐measures analyses (Friedman *Q* test) revealed no statistically significant differences over time (*p* > 0.05). The *Q*‐test was not applicable for the CD8+ subpopulation.

### 3.3 RT‐qPCR

Overall, RT‐qPCR detected SARS‐CoV‐2 RNA in 21 of 38 patients (55.3%). When analyzing individual specimens, the virus was detected in 23 of 57 semen samples (40.4% overall). This detection rate was highly dependent on the assay protocol: the initial E‐gene protocol identified only 1 positive out of 15 samples tested (6.7%), whereas the optimized, high‐sensitivity N1‐gene protocol detected viral RNA in 22 of 42 samples (52.4%). All positive cases exhibited high Ct values, ranging from 38 to 39, which indicates viral loads near the assay's LOD.

In the mild COVID‐19 group, 11 of 27 patients had at least one positive sample. Two tested positive only in the second collection, while three were positive in the third, with two of them having no prior detection of SARS‐CoV‐2 RNA (Figure 2A). In the moderate group, 91% (10/11) of patients had detectable SARS‐CoV‐2 in semen (Figure 2B). Among 21 samples, most underwent replicate testing. Consistent with the stochastic nature of detection at the LOD, seven of the ten positive patients in this group showed discordant replicates (one positive and one negative), while three were concordant (Figure 2B). To ensure that these borderline signals represented true low‐level viral presence rather than technical artifacts, the positive samples were subjected to further validation via viral isolation in the Vero cell culture.

**FIGURE 2:**
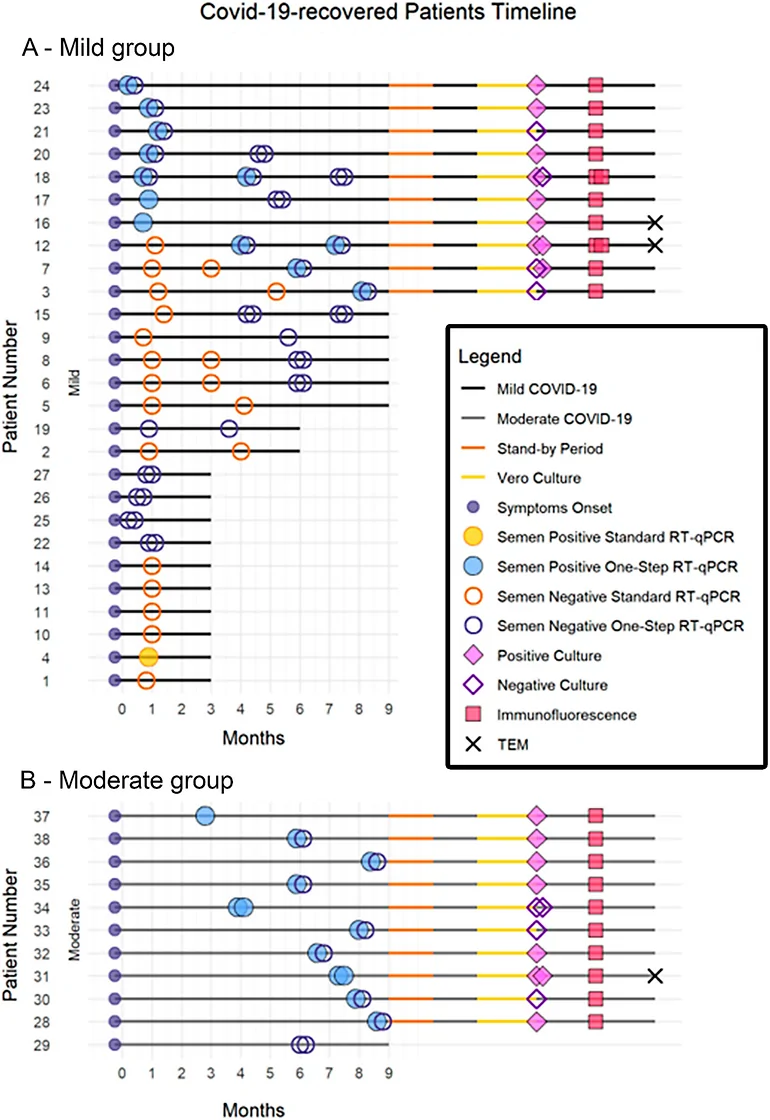
Timeline of study design and sampling intervals for the evaluated groups. The schematic illustrates the temporal distribution of semen collection for participants from the initial SARS‐CoV‐2 diagnosis through the post‐acute phase to investigate viral persistence and potential infectiousness. (A) Longitudinal follow‐up of the mild COVID‐19 group (*n* = 27, lines), with sampling points at approximately 1, 3, and 6 months post‐recovery (circles). (B) Cross‐sectional assessment of the moderate COVID‐19 group (*n* = 11, lines), highlighting the single time point of semen collection (circles). RT‐qPCR positive samples were further investigated using Vero CCL‐81 cell culture (rhombus) to assess replication, immunofluorescence to detect the S protein (squares), and transmission electron microscopy (TEM) (X symbol) to identify the morphology of viral particles. To map the Patient Numbers (1–38) used in this timeline to their corresponding semen sample IDs utilized in Table 4 and Table S3, please refer to the sample‐to‐patient mapping key provided in Table S4.

The vaccination rate was lower in the mild group and higher in the moderate group (Table S1). The vaccination status of patients was not associated with SARS‐CoV‐2 detection in semen (Table S2). However, due to our small‐sample size and the vaccination imbalance between groups (Table S1), this analysis is underpowered, representing a lack of statistically significant association rather than a definitive absence of effect.

### 3.4 Genotyping

To distinguish reinfection from viral persistence, we attempted to genotype all RT‐qPCR‐positive semen samples to compare the results with the variants circulating during the patients' initial infection period, which led to their recruitment for this study. Unfortunately, the overall viral recovery and RNA integrity in our protocol were still insufficient for consistent whole‐genome sequencing across most specimens. Only sample 66 yielded sufficient coverage (65.2%) and depth (18.8%) and was identified as lineage 19A (clade B) by Nextclade; however, Pangolin could not assign a lineage. The consensus FASTA sequence for Sample #66 is provided in File S1 (EPI_ISL_20531814).

### 3.5 Vero Cell Data

To assess potential viral activity in culture, Vero CCL‐81 cell cultures were inoculated under BSL‐3 conditions with 24 specimens. These represented 12 positive specimens from the mildly ill group (representing 10 patients, after excluding the single E‐gene‐positive sample to maintain protocol standardization) and 12 positive specimens from the moderately ill group (representing 10 patients, including parallel duplicate aliquots from two patients to evaluate culture reproducibility). A limited increase in viral copies was observed in mild and moderate COVID‐19 samples. Observing at least 2 time points in the Vero cell culture supernatant, the Ct value decreased in 6 mild samples and 1 moderate sample (Table S3). Notably, as illustrated in Figure 3A, viral load tended to plateau after 24–48 h in most samples, suggesting a restricted in vitro replication potential or low‐level viral persistence (Figure 3 and Table S3).

**FIGURE 3:**
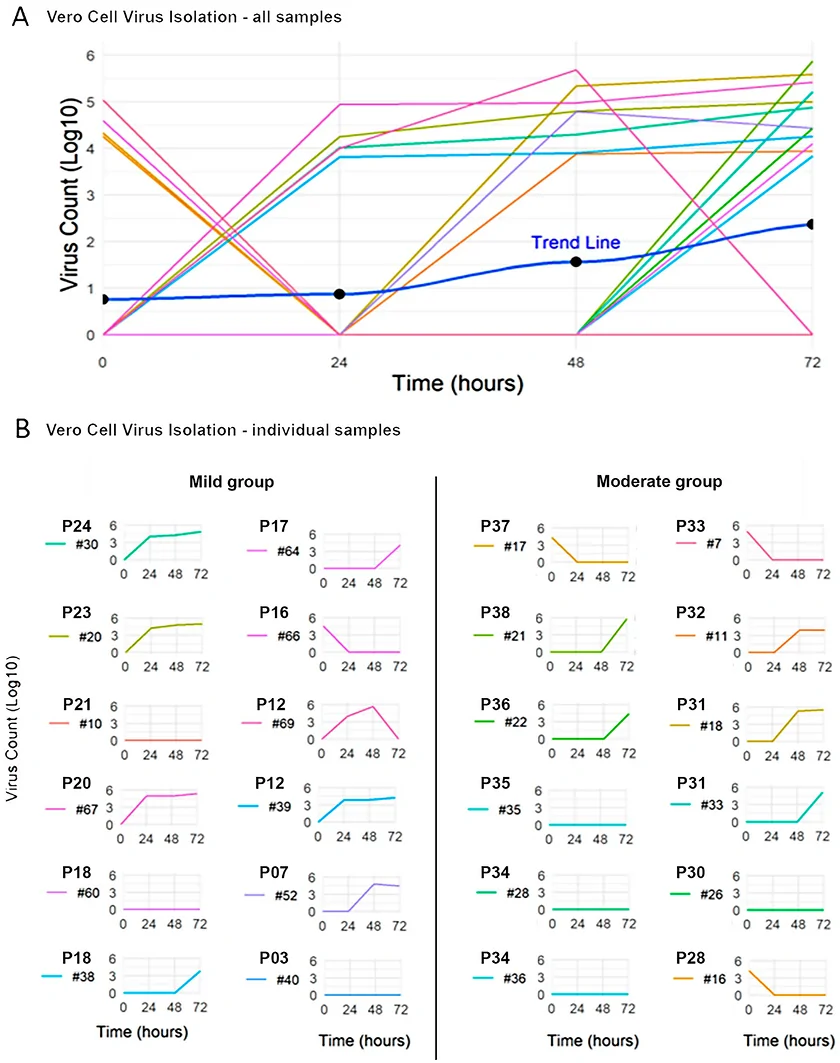
Evaluation of potential SARS‐CoV‐2 kinetics in Vero CCL‐81 cell cultures. (A) Superimposed viral load trajectories (Log_10_ RNA copies/mL) over 72 h for the evaluated specimens, with the bold blue line representing the overall mean trend. (B) Individual kinetic profiles of the 24 specimens evaluated, comprising 12 specimens from the mildly ill group and 12 from the moderately ill group (including duplicate parallel specimens from two patients to observe reproducibility). Note that most specimens exhibited a restricted kinetic profile, with viral copy numbers peaking or plateauing between 24 and 48 h, suggesting low‐level persistence or restricted in vitro replication potential. Absolute viral counts were calculated from Ct values using a standard curve.

Despite this limited expansion, all 24 samples showed S protein by immunofluorescence. Furthermore, three samples with the highest fluorescence intensity, collected at 21, 123, and 222 days post‐infection (corresponding to approximately 1, 3, and 6 months, respectively), were analyzed by TEM, revealing structures morphologically compatible with coronavirus‐like particles within endosomal vesicles in the Vero cell cytoplasm (Figures 4 and 5 and Table 4). In the absence of immunogold labeling, these ultrastructural findings were interpreted with caution and considered supportive rather than definitive evidence of infection. The complete integration of individual clinical timelines, laboratory sample codes, and multi‐method results is compiled in Table S4 (S4a for mild and S4b for moderate cohorts).

**FIGURE 4:**
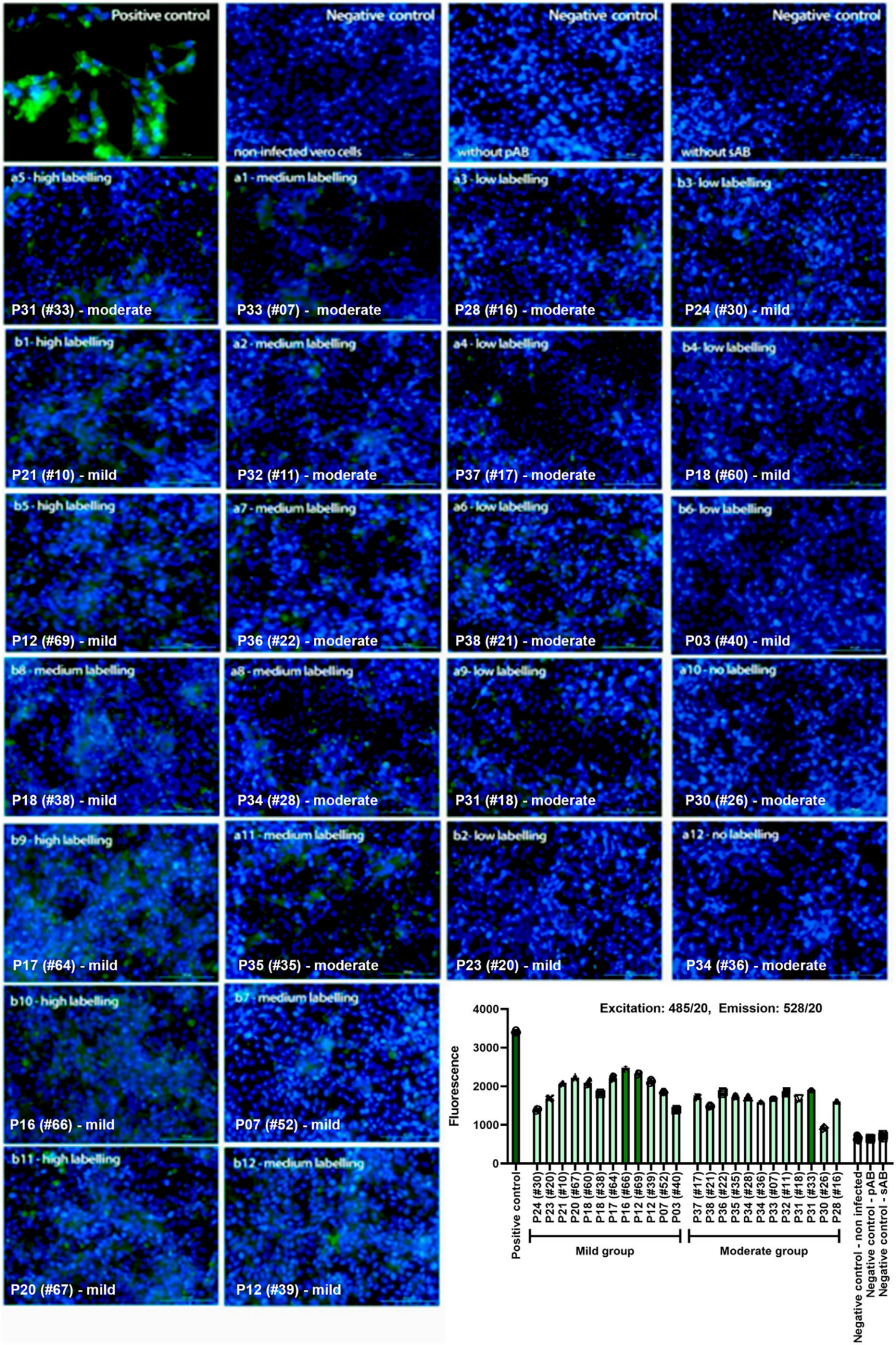
Evaluation of SARS‐CoV‐2 Spike (S) protein by immunofluorescence in Vero CCL‐81 cell cultures. The panel shows representative fluorescence micrographs of cell cultures 24 h after inoculation with positive control (live SARS‐CoV‐2 Delta strain), negative controls (uninfected Vero cells, without primary antibody, and without secondary antibody), and the 24 evaluated specimens. These specimens comprise the 24 evaluated specimens, consisting of 12 positive specimens from the mildly ill cohort (representing 10 patients) and 12 positive specimens from the moderately ill cohort (representing 10 patients, including parallel duplicate aliquots to assess culture reproducibility). Micrographs are labeled by specimen identifier and qualitative labeling level (high, medium, low, or no labeling). The inset bar chart depicts the quantitative fluorescence intensity (excitation: 485/20 nm, emission: 528/20 nm) across all groups. In accordance with the restricted viral kinetics observed and the strong signal in the positive control group, these signals represent low‐level viral antigen presence in culture from the mild and moderate group. The samples P16 (#66), P12 (#69), and P31 (#33) (highlighted as dark green bars in the chart) were selected for ultrastructural TEM analysis. Nuclei are stained with DAPI (blue) and SARS‐CoV‐2 S protein is stained with Alexa Fluor 488 (green). Scale bars = 100 µm. TEM, transmission electron microscopy.

**FIGURE 5:**
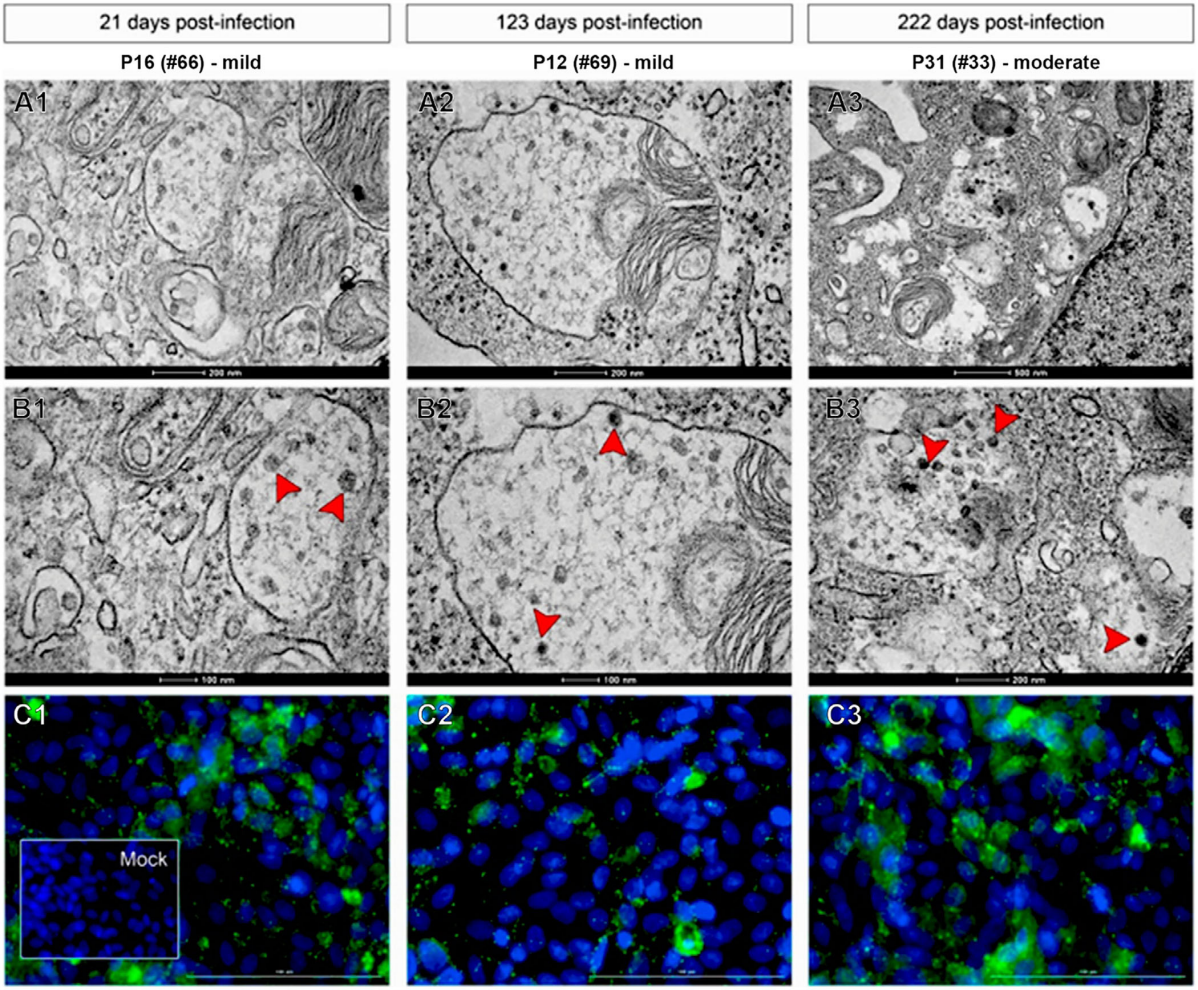
Ultrastructural TEM analysis and immunofluorescence of infected Vero cells. (A1–A3) TEM of three representative patient specimens selected based on having the highest fluorescence intensity measured by microplate fluorometry, representing the three collection windows at 21, 123, and 222 days post‐infection (corresponding to approximately 1, 3, and 6 months, respectively). Panels (B1–B3) depict a higher magnification of the corresponding fields from row A; red arrowheads indicate structures morphologically compatible with SARS‐CoV‐2 viral particles inside cytoplasmic endosomal vesicles in Vero cells. Panels (C1–C3) show the corresponding immunofluorescence assay for the SARS‐CoV‐2 spike protein of the same specimens. The inset in panel (C1) displays uninfected Vero cells (Mock control) processed under identical conditions. TEM, transmission electron microscopy.

**TABLE 4:** Fluorescence intensity according to the Vero cells immunofluorescence.

Sample	P24 (#30) (mild)	P23 (#20) (mild)	P21 (#10) (mild)	P20 (#67) (mild)	P18 (#60) (mild)	P18 (#38) (mild)
Value 1	1606.00	1845.00	1893.00	2205.00	2119.00	1843.00
Value 2	1581.00	1799.00	1791.00	2259.00	2084.00	1798.00
Value 3	1566.00	1873.00	1891.00	2200.00	2070.00	1793.00

Sample	P17 (#64) (mild)	P16 (#66) (mild)	P12 (#69) (mild)	P12 (#39) (mild)	P07 (#52) (mild)	P03 (#40) (mild)
Value 1	2217.00	2536.00	2355.00	2077.00	1876.00	1418.00
Value 2	2193.00	2431.00	2281.00	2170.00	1839.00	1393.00
Value 3	2235.00	2480.00	2348.00	2128.00	1828.00	1367.00

Sample	P37 (#17) (mod.)	P38 (#21) (mod.)	P36 (#22) (mod.)	P35 (#35) (mod.)	P34 (#28) (mod.)	P34 (#36) (mod.)
Value 1	1894.00	1726.00	1700.00	1701.00	1716.00	1409.00
Value 2	1894.00	1709.00	1668.00	1668.00	1773.00	1357.00
Value 3	1895.00	1707.00	1689.00	1699.00	1707.00	1415.00

Sample	P33 (#07) (mod.)	P32 (#11) (mod.)	P31 (#18) (mod.)	P31 (#33) (mod.)	P30 (#26) (mod.)	P28 (#16) (mod.)
Value 1	1680.00	1618.00	1481.00	2044.00	925.00	1736.00
Value 2	1675.00	1605.00	1513.00	2069.00	953.00	1713.00
Value 3	1676.00	1609.00	1469.00	2079.00	892.00	1682.00

*Note*:

* The samples with the highest intensity for each time period (1, 3, or 6 months) are highlighted in blue. Those samples were taken to the TEM.

## 4 Discussion

Here, we aimed to determine whether SARS‐CoV‐2 could be detected in the semen of mild and moderate COVID‐19‐recovered patients and to assess how long the virus persists, with follow‐up up to 6 months after symptom onset. We found SARS‐CoV‐2 genetic material (RNA) in semen from 11/27 mild COVID‐19‐recovered patients and 10/11 moderate ones. Furthermore, we detected persistent viral RNA and associated culture‐derived replication signals up to 8 months after the onset of symptoms using complementary techniques. These findings suggest that low‐level viral RNA and replication‐associated signals may persist for a prolonged period in the MGT, pointing to the MGT as a potential biological reservoir after clinical recovery.

Our study shows a temporary decline in sperm quality after symptomatic COVID‐19, with recovery between 3 and 6 months post‐infection. However, it is important to note that in the mild group, only 7 patients contributed all three samples for the longitudinal study. This small‐sample size hampered statistical power; hence, these recovery pattern data should be considered exploratory and hypothesis‐generating rather than confirmatory. Nonetheless, the observed recovery timeline suggests that more than one spermatogenic cycle is necessary to recover sperm parameters. This may be due to direct testicular injury, inflammation, immune or hormonal disruption, fever, medication, or stress—all known reversible factors affecting spermatogenesis. These factors may impair spermatogenesis individually or collectively, though such effects are typically reversible.

Multiple studies report COVID‐19‐related declines in semen volume, concentration, motility, morphology, and viability, regardless of disease severity [9, 11, 12, 13, 14, 15, 16, 17, 18, 19, 20, 21, 22, 23, 24, 25, 26, 27, 28, 29, 30, 31, 32, 33]. Most show deterioration within 3 months and recovery by 6 months [11, 17, 19, 20, 21, 24, 28, 29, 32, 33], consistent with our results. Others found no impact [34, 35, 36, 37, 38, 39, 40, 41, 42, 43], and only Best et al. (2021) reported persistent impairment during a 91‐day follow‐up [14]. Longitudinal data suggest recovery after 1 or 2 spermatogenic cycles, with semen parameters generally remaining above the fifth WHO percentile, including in our cohort. We also observed transient seminal inflammation, with CD3, CD4, CD8, and CD45+ cells peaking at 3 months and returning to normal by 6 months [9, 11, 28, 44]. It is necessary to report that we did not detail inflammatory mediators (such as cytokines) or lymphocyte activation markers, which would provide deeper insights into the local immunological environment. However, our lymphocytic data are consistent with increased seminal cytokines post‐COVID‐19, as reported by Martinez et al. (2023), who found inflammation lasting up to 3 months [28].

We detected SARS‐CoV‐2 genetic material (RNA) in the semen of 21 out of 38 (55%) mildly to moderately ill recovered patients by RT‐qPCR, with presence/persistence for over 6 months in some cases. While our exploratory analysis showed no statistically significant association of vaccination status with this viral detection, this subanalysis is underpowered due to our small‐sample size and severity‐related vaccination differences between groups. Among 37 previous studies, only six reported viral presence in semen at lower rates (2%–16%) [5, 6, 7, 8, 9, 10]. Most of these studies evaluated patients during the acute or early recovery phases, with varying degrees of illness severity (mild, moderate, and severe). In contrast, our study focused solely on mild and moderate recovered cases. Notably, Purpura et al. (2022) are an exception, detecting viruses in a similar recovery context [10].

On the other hand, the remaining 31 studies did not detect SARS‐CoV‐2 RNA in semen [11, 12, 13, 14, 15, 16, 17, 18, 19, 20, 21, 34, 35, 36, 37, 38, 39, 40, 43, 44, 45, 46, 47, 48, 49, 50, 51, 52, 53, 54, 55]. Of these, 13 studies focused on patients in the acute phase of infection (≤15 days) and included asymptomatic and mildly, moderately, and severely symptomatic individuals [11, 18, 20, 35, 36, 38, 39, 45, 46, 49, 50, 52, 53]. Even long‐term studies, such as those conducted by Pavone et al. (2022) and Lauritsen et al. (2024), which followed patients for up to 30 days and 3–6 months, respectively, did not detect the virus in semen [20, 53]. The other 18 negative studies also included asymptomatic and symptomatic COVID‐19 patients of varying severity [12, 13, 14, 15, 16, 17, 19, 21, 34, 37, 40, 43, 44, 47, 48, 51, 54, 56], and 11 of these studies specifically tested semen samples collected 30 days or more after the onset of symptoms [13, 14, 15, 16, 17, 34, 40, 43, 44, 48, 51].

Interestingly, despite the lack of detectable virus (RNA) in semen in the majority of the studies, one report identified viral particles in spermatozoa using TEM in 9 out of 13 patients (69%) up to 90 days after hospital discharge—corresponding to approximately 100 to 110 days post‐infection [54]. In a separate study, viral RNA fragments were detected in 2 out of 12 patients (17%) using a capture‐based probe hybridization method [40]. These findings suggest that, while infectious virus may not be consistently present in semen, viral components can persist in spermatozoa for extended periods after clinical recovery.

Several studies, including the one by Li et al. (2020) [5], have faced criticism due to methodological shortcomings that may have contributed to false‐positive RT‐qPCR results in semen [56]. Their study included only severe COVID‐19 cases, which are known to be associated with higher systemic viral loads and, potentially, a greater likelihood of viral dissemination to body fluids like semen [56]. Moreover, false positives in RT‐qPCR testing can arise due to several factors: contamination, poor test specificity, and improper determination of the threshold at which a sample with a low viral load is considered truly positive [55, 57]. In fact, they did not report key parameters, such as Ct values, primers used, or the LOD, nor did they clarify whether aseptic techniques were used during sample collection [5].

Subsequent positive studies, including ours, addressed these concerns by implementing stricter protocols to avoid semen contamination [6, 7, 8, 10]. However, we acknowledge that microbiological profiling to definitively exclude bacterial contamination was not performed in our protocol. Another potential source of semen contamination is residual viral shedding from the urinary tract [7, 16, 56]. However, Saylam et al. (2021) mitigated this risk by collecting semen and urine samples at separate times to prevent cross‐contamination. In their study, the only positive semen sample showed no viral presence in urine, confirming the specificity of their RT‐qPCR results [7]. Importantly, blood or saliva were not collected in our study. Future longitudinal studies should incorporate multi‐site sampling to clarify the compartmentalization of possible SARS‐CoV‐2 reservoirs.

A recurring concern in studies reporting positive RT‐qPCR results in semen is the detection of SARS‐CoV‐2 at late‐cycle thresholds (Ct 38–39), which some interpret as false positives. However, analytical validation of RT‐qPCR assays ensures high specificity, and Ct values of 40 or less are widely accepted as positive [58]. In our study, we detected the virus at late Ct values (38–39 Ct) in all positive cases. These late Ct values likely reflect low viral RNA levels, especially near the assay's LOD, where test reproducibility is expected to be variable. At this threshold, the stochastic nature of viral distribution often leads to variable test reproducibility and discordant replicates, where a negative retest does not necessarily invalidate an initial positive signal [58]. These considerations may explain some of the discrepancies observed in our study and the inconsistent results reported in other research studies.

Multimodal validation was essential to overcome constraints imposed by borderline molecular signals. The identification of structures morphologically compatible with coronavirus‐like particles by TEM, alongside S‐protein presence, provides the necessary biological context to support these low‐level RT‐qPCR signals as true positives rather than artifacts. Supporting this interpretation, Hallak et al. (2024) found virus‐infected spermatozoa in nine of 13 patients using TEM despite negative RT‐PCR results. This discrepancy was attributed to the very low viral concentration in semen, which is plausible considering that the testes contribute only about 5% of the ejaculate [54]. Additionally, variations in SARS‐CoV‐2 viral load have been reported depending on the viral variant [58]. Another possible explanation for their findings is the lack of a viable virus with only the RNA‐free capsid present in the spermatozoa.

It is also important to acknowledge that RT‐qPCR, although the gold standard, is not infallible. Its sensitivity can vary from 70% to 98% depending on the test used, and false negatives can occur, particularly in samples with low viral loads or under suboptimal pre‐analytical conditions [59, 60]. False‐negative rates have been reported to be as high as 58% [61]. Factors such as improper sampling, RNA degradation, PCR inhibition, and sample mishandling (e.g., repeated freeze–thaw cycles) can significantly impact test performance, leading to late Ct values and false‐negative results [55].

Differences in study outcomes may also stem from variations in disease stage, symptom severity, timing of sample collection, potential contamination, and methodological inconsistencies. Notably, there is considerable heterogeneity in the RT‐PCR primers employed across studies. While most studies targeted common genomic regions (e.g., ORF1ab, N, S, E, RdRP), only 61% of the negative studies specified their primers [13, 16, 17, 18, 21, 36, 38, 39, 40, 43, 44, 45, 46, 48, 50, 51, 52, 53, 55], and less than half used high‐sensitivity targets such as the N gene and ORF1ab [13, 17, 18, 21, 36, 39, 40, 43, 44, 46, 48, 50, 52, 53]. Our own findings underscore the importance of primer selection; the transition from the E gene to the more sensitive N1 gene during our study was a deliberate technical optimization that proved crucial for detection, as 20 of our 21 positive cases were identified using the N1 target. Furthermore, only six of these studies described strategies for preserving RNA [18, 39, 40, 43, 46, 50]. Such variability in primer selection and sample‐handling protocols, particularly regarding the analytical sensitivity of different genomic targets, likely contributes to inconsistent SARS‐CoV‐2 detection rates in semen across studies.

To distinguish reinfection from viral persistence and provide definitive evidence regarding the source of the virus, we attempted to genotype all RT‐qPCR‐positive samples for comparison with the SARS‐CoV‐2 variants circulating during the patients’ initial infection period. Unfortunately, genotyping was inconclusive for most samples. Only one sample (Sample 66), collected 21 days post‐symptom onset, yielded sufficient coverage (65%) and was classified as lineage 19A—consistent with the variant circulating at the time of infection. Although genotyping could not definitively distinguish between persistence and reinfection, several findings suggest persistence. All patients were asymptomatic at the time of semen collection, and the likelihood of coincidental, asymptomatic reinfections in nearly all moderate cases between 4 and 9 months post‐infection is low. While not conclusive, these observations are compatible with the hypothesis that SARS‐CoV‐2 persists in the MGT (incorporating contributions from testes, epididymides, and accessory glands) following clinical recovery.

Lastly, it is crucial to understand that a positive molecular test detects viral RNA but not necessarily a viable virus. Confirming infectiousness requires isolating and culturing the virus. Although Ct values are often used as proxies for viral load or infectiousness, they are imperfect indicators of these measures. However, it remains unclear how many SARS‐CoV‐2 virions are required to cause infection or whether Ct values correlate reliably with the number of infectious virus particles. Some studies have even cultured viable SARS‐CoV‐2 from samples with fewer than the previously suggested 10^6^ genome copies [62].

Factors such as specimen quality, sample type, and laboratory conditions all influence Ct values and viral viability. These findings reinforce that we cannot entirely rely on Ct‐values as a direct measure of infectiousness [63]. Moreover, Ct values vary significantly between variants. For example, Omicron tends to show higher Ct values compared to earlier strains despite active infection, reinforcing the idea that Ct values alone cannot reliably indicate infectiousness [64]. While the presence of SARS‐CoV‐2 RNA could theoretically indicate non‐functional or ‘inert’ RNA, this is unlikely because such RNA is quickly broken down in the human body [65, 66]. In our study, all RT‐qPCR‐positive semen samples were cultured in Vero CCL‐81 cells to evaluate potential viral activity. Our culture data demonstrated restricted in vitro viral kinetics, characterized by a gradual decrease in Ct values that eventually stabilized. Rather than indicating high‐titer active replication, this pattern suggests low‐level, restricted viral persistence within the MGT.

Immunofluorescence confirmed the presence of the virus's S protein in all positive cultures. Three highly fluorescent samples (≤30 days, 3 months, and 6 months) were further analyzed by TEM, revealing structures morphologically compatible with coronavirus‐like particles within endosomal vesicles. Although the absence of immunogold labeling or successful whole‐genome sequencing precludes a definitive identification of these particles, the systematic application of strict ultrastructural criteria, such as size (60–140 nm), presence of an electron‐dense core, and surface projections, provides morphological support for the presence of viral material. To date, only three other studies have attempted semen culture in Vero cells [8, 36, 38]. However, despite detecting positive RT‐qPCR results in one patient in one study [8], none reported positive culture signals, further highlighting the low‐level, restricted nature of the viral persistence observed in our cohort.

This study has several important strengths. It is the first longitudinal investigation to monitor long‐term SARS‐CoV‐2 RNA clearance and associated culture signals in the semen of clinically recovered patients. By using complementary molecular, immunofluorescence, and ultrastructural approaches, our work addresses a key biological question regarding the existence of viral reservoirs in the MGT. While the clinical relationship between seminal viral persistence and symptomatic Long COVID remains a speculative hypothesis requiring validation in symptomatic cohorts, our translational data provide a valuable framework for understanding pathogen compartmentalization and its potential implications for male reproductive health.

However, some limitations must be acknowledged. The sample size, while in line with previous studies, was relatively small, and semen samples in the moderate disease group were obtained at a single time point, precluding assessment of intra‐individual variability and temporal trends. Although all parameters were within WHO reference ranges at the time of evaluation, these findings should be interpreted as cross‐sectional and descriptive, without inference regarding longitudinal changes or recovery. The absence of a control group, such as pre‐infection samples from the same patients, also precludes direct before‐and‐after comparisons of semen quality. Moreover, detailed clinical symptomatology, including fever, was not systematically captured, which is relevant since systemic illness may transiently affect spermatogenesis and represents a potential confounding factor. Importantly, as semen analysis was a secondary, exploratory outcome, these findings should be interpreted cautiously and without causal inference.

We also must acknowledge three key technical limitations. First, the absence of a concurrent cohort of COVID‐negative semen donors prevents us from empirically assessing the false‐positive rate of our late‐CT molecular signals in this complex matrix. Second, we were unable to obtain high‐quality consensus genome sequences from the semen due to very low viral loads. Third, since a live Delta strain was utilized as a positive control within the same BSL‐3 facility, the theoretical risk of cross‐contamination cannot be entirely ruled out. Although we mitigated this by enforcing strict biosafety protocols and running parallel negative controls (DMEM alone) that consistently showed no viral signal. Our culture‐derived observations should be conservatively interpreted as evidence of low‐level, restricted viral replicative activity within the MGT, rather than definitive confirmation of active patient‐derived infectiousness.

In conclusion, we detected SARS‐CoV‐2 RNA and low‐level replicative culture signals in the semen of patients recovered from mild to moderate COVID‐19. Our findings support a model of long‐term, restricted viral persistence in the MGT, characterized by low‐level, localized replicative dynamics rather than high‐titer active infection. While the clinical relevance of this persistent reservoir to Long COVID symptoms remains a hypothetical mechanism to be validated in future symptomatic cohorts, semen analysis represents a valuable non‐invasive tool for monitoring long‐term pathogen clearance and reservoir dynamics.

## Author Contributions

Marcelo H. Furtado, Bárbara P. Mendes, Juan C. González, Thiago P. Furtado, Samyra M. S. N. Lacerda, Guilherme M. J. Costa, were primarily responsible for the male reproductive aspects, sperm evaluation, and morphological data analysis. Fernanda R. Souza, Lidia M. Andrade, Anisleidy Pérez Castillo, and Renato S. Aguiar were responsible for SARS‐CoV‐2 genetic detection and Vero cell culture experiments. Individual contributions are as follows: MHF: conceptualization, data curation, literature search, investigation, methodology, project administration, figures, study design, data collection, data analysis, data interpretation, writing – original draft, and writing – review & editing; Lidia M. Andrade: investigation, methodology, figures, study design, data collection, data analysis, data interpretation; Renato S. Aguiar: study design, data analysis, data interpretation, and writing – review & editing; Fernanda R. Souza: investigation, methodology, data collection, data analysis, data interpretation; Anisleidy Pérez Castillo,: investigation, methodology, and data collection; Juan C. González,: writing – review & editing; Bárbara P. Mendes: data curation, investigation, methodology, project administration; Thiago P. Furtado: literature search, study design, data collection, writing – review & editing; SMSNL: conceptualization, data interpretation, and writing – review & editing; Guilherme M. J. Costa: conceptualization, data curation, literature search, investigation, methodology, project administration, figures, study design, data collection, data analysis, data interpretation, writing – original draft, and writing – review & editing.

## Funding

This study was funded by the Ferring COVID‐19 Investigational Grant (FIN0042393), with additional support provided to G.M.J.C. (FAPEMIG: APQ‐01078‐21, RED‐00079‐22; CNPq: 310946/2025‐5), SMSNL (FAPEMIG RED‐00135‐22), J.C.G. (RED‐00135‐22), and R.S.A. Instituto Todos pela Saúde—ITpS (C1294; C2024‐0779, A113); CNPq (315592/2021‐4, INCT‐One Health CNPq 405786/2022‐0); FAPEMIG (APQ‐02826‐24; APD‐00520‐25). The funders had no role in study design, data collection and analysis, decision to publish, or preparation of the manuscript.

## Conflicts of Interest

The authors declare no conflicts of interest.

## Supporting information




**Figure S1**. Flow cytometry gating strategy for the identification of semen lymphocytic subpopulations. (A) FSC‐H vs. SSC‐H density plot illustrating the gating of the leukocyte region (gated on FSC‐Height and SSC‐Height). (B) Selection of CD45+ leukocytes from the gated leukocyte population (FL4‐H:: CD45 APC). (C) Identification of T lymphocytes (CD3+) within the CD45+ leukocyte gate (FL1‐H:: CD3 FITC). (D) Dual‐parameter dot plot gating CD3+ T cells into CD4+ helper T cells (Q3) and CD8+ cytotoxic T cells (Q1) using FL3‐H:: CD4 PE‐Cy7 and FL2‐H:: CD8 PE, respectively. This representative gating strategy was utilized to perform the exploratory longitudinal and cross‐sectional analyses of semen immunological profiles.

**Supporting File 2**: andr70431‐sup‐0002‐S1.docx

**Supporting File 3**: andr70431‐sup‐0003‐S1.pdf

**Supporting File 4**: andr70431‐sup‐0004‐S2.pdf

**Supporting File 5**: andr70431‐sup‐0005‐S3.docx

**Supporting File 6**: andr70431‐sup‐0006‐S4.docx

## Data Availability

All data will be made available upon request to the corresponding author, subject to approval of a proposal and a signed data access agreement.
