# Supplementary material for: Detection of SARS‐CoV‐2 in Semen Months After COVID‐19: Potential Evidence of Low‐Level Replicative Persistence
Source: Andrology. 2026 Nov 9;15(1):e70431. doi: 10.1111/andr.70431 (PMC13748924; doi:10.1111/andr.70431)
Supplement: Supplementary file 1 [file ANDR-15-0-s001.pdf]

Table S1 - Vaccination status by group

Group	Non-vaccinated	Vaccinated
Mild	16/27 (59.2%)	11/27 (40.8%)
Moderate	2/11 (18.2%)	9/11 (81.8%)

Fisher's Exact test p= 0.033
