# Supplementary material for: Detection of SARS‐CoV‐2 in Semen Months After COVID‐19: Potential Evidence of Low‐Level Replicative Persistence
Source: Andrology. 2026 Nov 9;15(1):e70431. doi: 10.1111/andr.70431 (PMC13748924; doi:10.1111/andr.70431)
Supplement: Supplementary file 2 [file ANDR-15-0-s006.pdf]

Table S2 - SARS-CoV-2 detection in semen vs vaccination status by group

A - Mild group

Semen	Non-vaccinated	Vaccinated
PCR-	10/16 (62.5%)	6/16 (37.5%)
PCR+	6/11 (54.6%)	5/11 (45.4%)

Fisher's Exact test $p = 0.710$

B - Moderate group

Semen	Non-vaccinated	Vaccinated
PCR-	1/1 (100%)	0/1 (0.0%)
PCR+	1/10 (10.0%)	9/10 (90.0%)

Fisher's Exact test $p = 0.182$

C - All

Semen	Non-vaccinated	Vaccinated
PCR-	11/17 (64.7%)	6/17 (35.3%)
PCR+	7/21 (33.3%)	14/21 (66.7%)

Fisher's Exact test $p = 0.101$
