# Supplementary material for: Detection of SARS‐CoV‐2 in Semen Months After COVID‐19: Potential Evidence of Low‐Level Replicative Persistence
Source: Andrology. 2026 Nov 9;15(1):e70431. doi: 10.1111/andr.70431 (PMC13748924; doi:10.1111/andr.70431)
Supplement: Supplementary file 3 [file ANDR-15-0-s002.docx]

**Table S4a – Semen Sample-to-Patient Mapping Key for Mild COVID-19 Patients (Longitudinal Cohort)**

| **Patient ID** | **First Collection**  **(Semen Sample ID & RT-qPCR)** | **Second Collection**  **(Semen Sample ID & RT-qPCR)** | **Third Collection**  **(Semen Sample ID & RT-qPCR)** |
| --- | --- | --- | --- |
| Patient 24 | Sample #30 **(Positive, Ct 39) [Vero Culture + IF]**, Sample #1 (Negative) | - | - |
| Patient 23 | Sample #20 **(Positive, Ct 39) [Vero Culture + IF]**, Sample #5 (Negative) | - | - |
| Patient 21 | Sample #10 **(Positive, Ct 39) [Vero Culture + IF]**, Sample #14 (Negative) | - | - |
| Patient 20 | Sample #67 **(Positive, Ct 39) [Vero Culture + IF]**, Sample #63 (Negative) | Sample #24 (Negative), Sample #37 (Negative) | - |
| Patient 18 | Sample #60 **(Positive, Ct 38) [Vero Culture + IF]**, Sample #59 (Negative) | Sample #38 **(Positive, Ct 38) [Vero Culture + IF]**, Sample #55 (Negative) | Sample #19 (Negative), Sample #29 (Negative) |
| Patient 17 | Sample #64 **(Positive, Ct 39) [Vero Culture + IF]** | Sample #44 (Negative), Sample #47 (Negative) | - |
| Patient 16 | Sample #66 **(Positive, Ct 38) [Vero Culture + TEM (21 days) + Genotyped + IF]** | - | - |
| Patient 12 | Si 14 (Negative) | Sample #69 **(Positive, Ct 39) [Vero Culture + TEM (123 days) + IF]**, Sample #56 (Negative) | Sample #39 **(Positive, Ct 39) [Vero Culture + IF]**, Sample #61 (Negative) |
| Patient 7 | Si 11 (Negative) | Negative | Sample #52 **(Positive, Ct 39) [Vero Culture + IF]**, Sample #46 (Negative) |
| Patient 3 | Si 1 (Negative) | Si 2 (Negative) | Sample #40 **(Positive, Ct 39) [Vero Culture + IF]**, Sample #48 (Negative) |
| Patient 15 | Si 20 (Negative) | Sample #41 (Negative), Sample #54 (Negative) | Sample #57 (Negative), Sample #68 (Negative) |
| Patient 9 | Si 18 (Negative) | - | Sample #53 (Negative) |
| Patient 8 | Si 7 (Negative) | Si 8 (Negative) | Sample #43 (Negative), Sample #50 (Negative) |
| Patient 6 | Si 3 (Negative) | Si 4 (Negative) | Sample #49 (Negative), Sample #65 (Negative) |
| Patient 5 | Si 17 (Negative) | Si 19 (Negative) | - |
| Patient 19 | Sample #45 (Negative) | Sample #51 (Negative) | - |
| Patient 2 | Si 5 (Negative) | Si 6 (Negative) | - |
| Patient 27 | Sample #25 (Negative), Sample #4 (Negative) | - | - |
| Patient 26 | Sample #23 (Negative), Sample #9 (Negative) | - | - |
| Patient 25 | Sample #2 (Negative), Sample #31 (Negative) | - | - |
| Patient 22 | Sample #12 (Negative), Sample #8 (Negative) | - | - |
| Patient 14 | Si 10 (Negative) | - | - |
| Patient 13 | Si 13 (Negative) | - | - |
| Patient 11 | Si 16 (Negative) | - | - |
| Patient 10 | Si 15 (Negative) | - | - |
| Patient 4 | Si 12 **(Positive, Ct 39)** | - | - |
| Patient 1 | Si 9 (Negative) | - | - |

**Table S4b – Semen Sample-to-Patient Mapping Key for Moderate COVID-19 Patients (Cross-Sectional Cohort)**

| **Patient ID** | **Semen Collection**  **(Semen Sample ID & RT-qPCR)** |
| --- | --- |
| Patient 37 | Sample #17 **(Positive, Ct 38) [Vero Culture + IF]** |
| Patient 38 | Sample #21 **(Positive, Ct 39) [Vero Culture + IF]**, Sample #6 (Negative) |
| Patient 36 | Sample #22 **(Positive, Ct 39) [Vero Culture + IF]**, Sample #13 (Negative) |
| Patient 35 | Sample #35 **(Positive, Ct 39) [Vero Culture + IF]**, Sample #27 (Negative) |
| Patient 34 | Sample #28 **(Positive, Ct 39) [Vero Culture + IF]**, Sample #36 **(Positive, Ct 39) [Vero Culture + IF]** |
| Patient 33 | Sample #7 **(Positive, Ct 39) [Vero Culture + IF]**, Sample #3 (Negative) |
| Patient 32 | Sample #11 **(Positive, Ct 39) [Vero Culture + IF]**, Sample #34 (Negative) |
| Patient 31 | Sample #18 **(Positive, Ct 39) [Vero Culture + IF]**, Sample #33 **(Positive, Ct 39) [Vero Culture + IF Micrograph + TEM]** |
| Patient 30 | Sample #26 **(Positive, Ct 39) [Vero Culture + IF]**, Sample #15 (Negative) |
| Patient 28 | Sample #16 **(Positive, Ct 38) [Vero Culture + IF]**, Sample #32 (Negative) |
| Patient 29 | Sample #58 (Negative), Sample #70 (Negative) |
