# Supplementary material for: Detection of SARS‐CoV‐2 in Semen Months After COVID‐19: Potential Evidence of Low‐Level Replicative Persistence
Source: Andrology. 2026 Nov 9;15(1):e70431. doi: 10.1111/andr.70431 (PMC13748924; doi:10.1111/andr.70431)
Supplement: Supplementary file 5 [file ANDR-15-0-s005.docx]

|  | **RT-qPCR - semen** | | **RT-qPCR - Vero Cell Culture supernatant** | | | | | | | | | |
| --- | --- | --- | --- | --- | --- | --- | --- | --- | --- | --- | --- | --- |
| Sample | N1  (Ct) | 18S  (Ct) | Input – 0h  (Ct) | Input - 0h  (log_10_) | 24h  (Ct) | 24h  (log_10_) | 48h  (Ct) | 48h  (log_10_) | 72h  (Ct) | 72h  (log_10_) | Reducing Ct |  |
| P24 (#30) (Mild) | 39 | 20 | - | - | 28.06 | 1.04E+04 | 27.03 | 2.00E+04 | 24.92 | 7.65E+04 | + |  |
| P23 (#20) (Mild) | 39 | 19 | - | - | 27.21 | 1.79E+04 | 25.23 | 6.27E+04 | 24.50 | 1.00E+05 | + |  |
| P21 (#10) (Mild) | 39 | 18 | - | - | - | - | - | - | - | - |  |  |
| P20 (#67) (Mild) | 39 | 18 | - | - | 24.67 | 8.98E+04 | 24.59 | 9.45E+04 | 22.98 | 2.62E+05 | + |  |
| P18 (#60) (Mild) | 38 | 19 | - | - | - | - | - | - | - | - |  |  |
| P18 (#38) (Mild) | 38 | 16 | - | - | - | - | - | - | 28.72 | 6.85E+03 |  |  |
| P17 (#64) (Mild) | 39 | 18 | - | - | - | - | - | - | 27.72 | 1.29E+04 |  |  |
| P16 (#66) (Mild) | 38 | 19 | 25.95 | 3.97E+04 | - | - | - | - | - | - |  |  |
| P12 (#69) (Mild) | 39 | 17 | - | - | 28.13 | 9.95E+03 | 22.01 | 4.85E+05 | - | - | + |  |
| P12 (#39) (Mild) | 38 | 18 | - | - | 28.77 | 6.65E+03 | 28.47 | 8.04E+03 | 27.18 | 1.82E+04 | + |  |
| P07 (#52) (Mild) | 39 | 15 | - | - | - | - | 25.27 | 6.15E+04 | 26.53 | 2.75E+04 | + |  |
| P03 (#40) (Mild) | 39 | 18 | - | - | - | - | - | - | - | - |  |  |
| P37 (#17) (mod.) | 38 | 17 | 26.91 | 2.16E+04 | - | - | - | - | - | - |  |  |
| P38 (#21) (mod.) | 39 | 17 | - | - | - | - | - | - | 21.31 | 7.60E+05 |  |  |
| P36 (#22) (mod.) | 39 | 19 | - | - | - | - | - | - | 26.54 | 2.74E+04 |  |  |
| P35 (#35) (mod.) | 39 | 18 | - | - | - | - | - | - | - | - |  |  |
| P34 (#28) (mod.) | 39 | 20 | - | - | - | - | - | - | - | - |  |  |
| P34 (#36) (mod.) | 39 | 21 | - | - | - | - | - | - | - | - |  |  |
| P33 (#07) (mod.) | 39 | 19 | 24.36 | 1.09E+05 | - | - | - | - | - | - |  |  |
| P32 (#11) (mod.) | 39 | 18 | - | - | - | - | 28.57 | 7.52E+03 | 28.29 | 9.00E+03 | + |  |
| P31 (#18) (mod.) | 39 | 19 | - | - | - | - | 23.26 | 2.20E+05 | 22.36 | 3.90E+05 |  |  |
| P31 (#33) (mod.) | 39 | 16 | - | - | - | - | - | - | 23.69 | 1.67E+05 |  |  |
| P30 (#26) (mod.) | 39 | 21 | - | - | - | - | - | - | - | - |  |  |
| P28 (#16) (mod.) | 38 | 16 | 27.16 | 1.84E+04 | - | - | - | - | - | - |  |  |

**Table S3 – RT-qPCR Ct values in original semen samples and Ct values and viral load quantification (Log10) in Vero cell culture supernatants**
